# Performance evaluation in healthcare: the experience of maternity pathway from Tuscany to the Italian network of regions

**DOI:** 10.1186/1824-7288-40-S1-A35

**Published:** 2014-08-11

**Authors:** Manila Bonciani, Barbara Lupi, Sabina Nuti

**Affiliations:** 1 Laboratorio Management e Sanità, Istituto di Management, Scuola Superiore Sant’Anna Pisa, Italy

## Background

Considering that performance measurement is largely recognised as a key tool for quality improvement, which kind of performance evaluation can be useful for healthcare professional, general managers and regional policy makers to improve the mother and child healthcare services? The presentation of the experience in assessing the maternity pathway (MP), made initially by Tuscany and successively shared within a network of Italian regions, will contribute to answer this question.

## Materials and methods

Since 2004, within the general framework of the multidimensional performance evaluation system introduced in Tuscany to assess and monitor local health authorities and teaching hospitals [1], a specific MP performance measurement system (PMSMP) was adopted in order to evaluate the MP process and outcomes [2]. It was based on several indicators grouping in six dimensions: population’s state of health, compliance with regional guidelines, efficiency and financial performance, clinical and health assessment, patient satisfaction (assessing the women’s experience of MP care), employees’ satisfaction (measuring the MP organizational climate). Starting from 2008 this evaluation system has been implemented also by a network of Italian regions (Basilicata, Autonomous Provinces of Bolzano and Trento, Liguria, Marche, , Umbria, Veneto and recently also Emilia Romagna and Friuli Venezia Giulia) [3].

## Results

The PMSMP allowed to monitor the progressive changes in the Tuscan and other regional MP. Its implementation during these years have showed that the benchmarking approach encouraged to learn from other experiences, by identifying best practices in mother and child healthcare services. The mechanism of scoring the evaluation indicators and displaying them on a spider diagram allowed to identify immediately the performance dimensions with weak results requiring specific strategies for improvement. Actually, the PMSMP is developing in order to answer the regional and local health authorities managerial needs. A regional professional group is discussing on the introduction of specific indicators on healthcare process and results of new-born infants, and at the level of the Italian regions network, new indicators are being identified in order to evaluate specifically the paediatric care at hospital level.

## Conclusions

The experience developed in Tuscany and in the network of the Italian regions represents a good example of performance evaluation in healthcare, also for the involvement of health professionals in identifying and defining evaluation indicators. It is an useful tool to evaluate all the phases of MP (pregnancy, delivery and postpartum) with managerial purposes, by identifying the organisational determinants of the MP process and outcomes.
